# Aberrant NDRG1 methylation associated with its decreased expression and clinicopathological significance in breast cancer

**DOI:** 10.1186/1423-0127-20-52

**Published:** 2013-07-30

**Authors:** Lin-Lin Han, Lin Hou, Ming-Jin Zhou, Zhong-liang Ma, Dong-Liang Lin, Li Wu, Yin-lin Ge

**Affiliations:** 1 Department of Biochemistry and Molecular Biology, Medical College, Qingdao University, Qingdao 266021, China; 2 Department of Clinical Laboratory, Qilu hospital of Shandong University, Jinan, Shandong 250012, China; 3 Department of Breast Surgery, the Affiliated Hospital of Qingdao University Medical College, Qingdao, China; 4 Department of Pathology, the Affiliated Hospital of Qingdao University Medical College, Qingdao, China

**Keywords:** Breast cancer, DNA methylation, Gene expression, N-myc downstream regulated gene

## Abstract

**Background:**

Cancer cell differentiation is an important characteristic of malignant tumor and has a great impact on prognosis and therapeutic decision for patients. The N-myc downstream regulated gene 1 (NDRG1), a putative tumor suppression gene, is involved in the regulation of human cell differentiation and metastasis in various cancers. Changes in the status of methylation of the NDRG1 gene have not been studied in detail in human breast cancer.

**Results:**

The MDA-MB-231 breast tumor cell line could express NDRG1. However, it was only expressed after treatment with 5-Aza-2*'*-deoxycytidine (AZA) in T47D cell line, which revealed that NDRG1 expression could modulated by DNA methylation. Therefore, the fragment surrounding the transcript start site of NDRG1 gene promoter was cloned after sodium bisulfite DNA treatment. A high density (66%) of methylation for human NDRG1 gene promoter was detected in T47D; however, there was only 16% of methylated CpG dinucleotides in the NDRG1 gene promoter in MDA-MB-231. DNA methylation in the NDRG1 promoter was detected in 31.1% of primary breast cancer samples. Furthermore, the NDRG1 promoter methylation correlated with the Tumor Node Metastasis (TNM) at stage III/IV, metastasis, lymph invasion, moderate and poor histological grade in the breast cancer patients.

**Conclusion:**

These findings suggest that the DNA methylation status of NDRG1 gene may play an important role in the pathogenesis and/or development of breast cancer, and the expression could be regulated by aberrant DNA methylation.

## Background

Breast cancer is one of the most common malignancies in women and is considered a genetic disease [1]. Like other cancers, it is involved in tumor occurrence and development because it influences oncogene and tumor suppressor gene expression as well as genomic stability. N-myc downstream regulated gene 1 (NDRG1), which has been mapped to the human chromosome 8q24.2, has a cDNA length of 1182 bp and encodes a cytoplasmic 43 kD protein. Previous studies have shown that NDRG1 expression is down-regulated in various cancer tissues, such as colon, prostate, and breast cancer tissues [2-4]. Accordingly, Kurdistani et al. [5] also reported that NDRG1 was downregulated in various cancer cell lines, and the growth of tumor cells is inhibited when the tumor cell line is transfected with NDRG1 cDNA. Meanwhile, low expression of NDRG1 is liked to result in a poor clinical outcome in breast cancer [3]. Together, these results suggest that NDRG1 is a tumor suppressor gene in various human cancers and the mechanism of NDRG1 gene expression loss needs to be investigated.

One way in which cancer cells depress tumor suppressor gene expression is through epigenetic alterations, which include DNA hypermethylation of the promoter CpG islands [6,7]. Genes with an abnormal methylation status, silenced by hypermethylation or activated by demethylation, are important for the development [8], progression [9], and cellular differentiation of tumors [10]. N-myc downstream regulated gene (NDRG1) was described as a potential tumor suppressor gene in various human cancers, and could be associated with tumor aggressiveness and metastasis [11-13]. Recent research found that NDRG1 is upregulated in gastric, colon and pancreatic cancer cells through demethylation of the CpG loci in the promoter region [14,15], suggesting that the epigenetic regulation mechanism plays an important role in activating NDRG1 expression during cancer progression and gives cancer cells a changeable trait. Interestingly, NDRG1 was revealed in the existence of CpG islands through the computational analysis of its promoter region by  Meth-Primer  (http://www.urogene.org/methprimer/index1.html). However, in studies of breast cancer tissues, the methylation status of NDRG1 has not been reported.

In the present study, we first examined the relationship between the methylation status of the NDRG1 gene promoter and its steady-state expression in breast tumors, corresponding normal tissues and two breast cancer cell lines. Using bisulfite sequencing, as well as nested-methylation-specific PCR (nested-MSP), we further analyzed the different NDRG1 methylation status in these two breast cancer cell lines. The methylation status in the breast tumor samples was also detected and the association with the clinicopathological data was analyzed to further explore the relationship between the methylation status of NDRG1 gene and the development of breast cancer.

## Methods

### Tissue sample collection

All tissue samples were obtained from the surgical specimens of patients who underwent mastectomy at the affiliated hospital of Qingdao university. All patients provided informed consent, and all procedures were approved by the hospital’s ethics board. The collected tissue samples were taken form unrelated Chinese women, aged 26 to 86 years (mean 52.3 ± 10.6), with sporadic breast cancer; there were 389 samples (302 paraffin embedding tissues and 87 fresh frozen tissues) containing >50% tumor area. Meanwhile, corresponding fresh-frozen normal tissues from the same patient, located at least 5 cm away from the tumor site, were also collected. These tissues were collected after reconfirmation by a senior pathologist from the Affiliated Hospital of Qingdao University. The histological grades of each tumor was determined according to the modified Bloom-Richardson criteria, and the Tumor Node Metastasis (TNM) stage was determined with the official classification methods [16].

### Cell culture and treatment with 5-Aza-2′-deoxycytidine (AZA)

Human breast cancer cell lines MDA-MB-231 and T47D were maintained in RPMI 1640 (GIBCO BRL, Grand Island, NY, USA) and supplemented with 10% fetal bovine serum (Hyclone, Logan, UT) at 37°C in the humidified atmosphere with 5% CO_2._ After serum starvation for 24 hours, two breast cancer cell lines were seeded at 10^5^ cells in four-well plates, allowed to attach for 24 hours and treated with 0, 2.5, 5 and 10 μM of the de-methylating agent, AZA.

### Reverse transcription (RT) PCR assay

Total RNA was extracted from breast cell lines and cDNA was prepared by RT-PCR kit (promega) according to the instructions. The primer used was *NDRG1*: 5′-ACTCCTCTGGAAAGACTTGTGC-3′ and 5′-AGTTGGGAGGAGGAAGTAGTCC3′ (D87953), 175 bp. *GAPDH* was used as a internal control: 5′-CAAGGTCATCCATGACAACTTTG-3′ and 5′-GTCCACCACCCTGTTGCTGTAG-3′, 491 bp. The PCR products were subjected to gel electrophoresis in 2% agarose gels.

### Protein extraction and western blotting

Total protein from MDA-MB-231 and T47D cell lines were extracted with standard method. The protein concentration was routinely analyzed by 12% SDS-PAGE. Protein was transferred to nitrocellulose membrane, which was blocked with 5% non-fat dry milk and incubated with rabbit anti-NDRG1 primary antibody (Zymed, San Francisco, CA) overnight at 4°C. The secondary horseradish peroxidase-conjugated anti-rabbit antibody was visualized with SuperSignal West Pico Chemiluminescent Substrate (Thermo Scientific, US). Equal protein loading was confirmed through β-actin.

### Sodium bisulfite sequencing analysis and nested-MSP

The genomic tumor DNA was isolated from the MDA-MB-231 and T47D breast cancer cell lines or tissue samples using phenol-chloroform. The DNA was then converted from unmethylated cytosines into uracils by bisulfite with EZ DNA Methylation-Gold Kit (Zymo Research, Orange, CA, USA) according to the manufacturer’s instructions. Previous studies have revealed that the region surrounding the transcription start sites (+1) of genes may regulate their expression [17]. We elected the region from −379 to +86 as target fragment. The primers were analyzed by Methyl Primer Express v1.0 software (http://www.appliedbiosystems.com/absite/us/ en/home/support/software-community/free-ab-software.html) using GenBank NM_006096 (NDRG1) as the reference sequence (outside primer: sense, 5′ -AGAAATTTTGAGGTAGAGATGGG-3′, antisense, 5′-ACTTCAACACCAACTAAAAACCA-3′, 465 bp; methylated primer: sense, 5′-GGGTTTCGATGTTTTTTTCGGC-3′, antisense, 5′-GCAATCCCAACACGC AACCGAAA-3′, 170 bp; unmethylated primer: sense, 5′-GGGTTTTGATGTTTTTTTTGGT-3′ ; antisense, 5′ -ACAATCCCAACACACAACCAA-3′, 170 bp). First round amplifications were performed in 25 uL reactions using perfectshot Taq (Takara), 10 pmol of outsider primer and 30 ng modified DNA and the following cycle parameters: 95°C for 5 min, then 35 cycles of 95°C for 30 s, 52°C for 40 s, 72°C for 40 s, followed by a final extension of 72°C for 5 min. Aliquots of 1 uL PCR products were subjected to second round amplification using methylated and unmethylated primer, respectively. The following cycle parameter: 95°C for 5 min, the 30 cycles of 95°C for 30 s, 57°C for 30 s, 72°C for 30 s, followed by a final extension of 72°C for 7 min.

### Statistical analyses

Promoter methylation of NDRG1 was analyzed using Chi-squared test by SPSS 13.0. The corelation of the clinicopathological data using unconditional logistic regression was used to estimate odds ratios (ORs) and 95% confidence intervals (CIs). All models were adjusted for age at diagnosis. Differences were considered statistically significant at P < 0.05.

## Results

### NDRG1 mRNA expression in breast tumors and corresponding normal tissues

**Figure 1:**
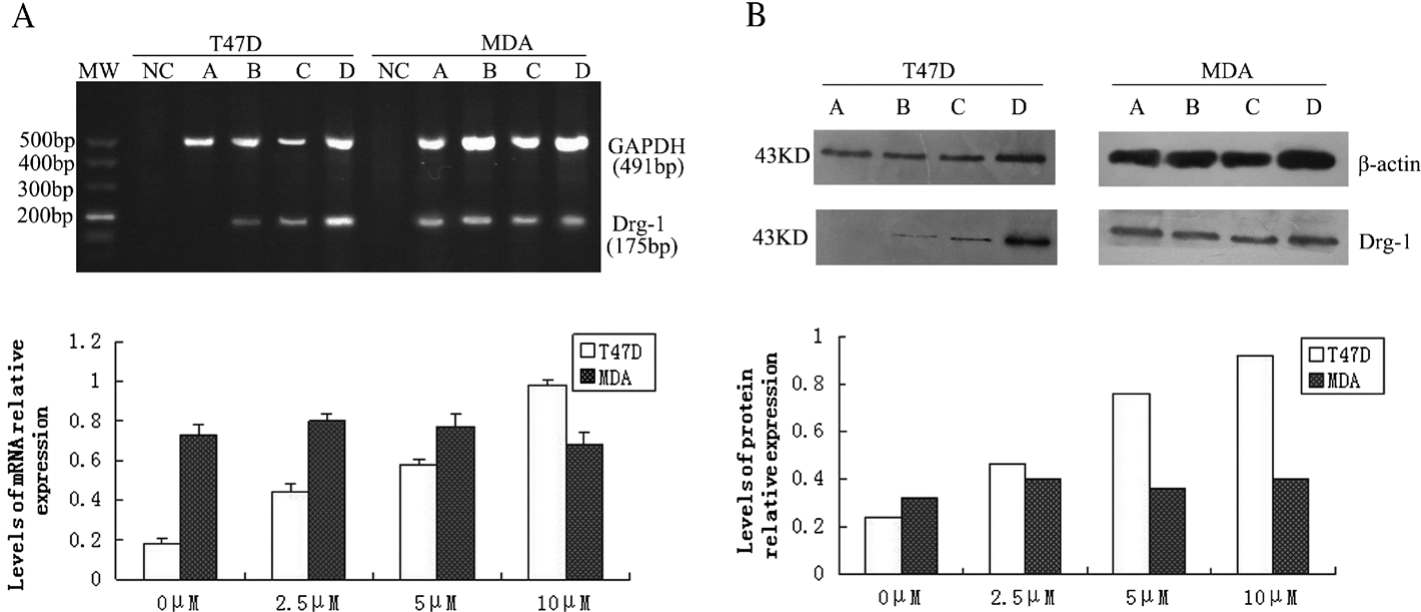
**NDRG1 mRNA expression analysis in breast tumor tissues (T) and corresponding adjacent tissues (N). (A)** NC represents the PCR reaction without DNA. The GAPDH gene was used as internal control. M, Molecular Weight. **(B)** The PCR products were scanned and analyzed using the Peiqing JS-380 Gel Imaging Analysis System after agarose electrophoresis. The integral absorbance (IA) score for each band was derived from formula IA = average absorbance × area, and the NDRG1 relative expression of each sample was represented in the following way: NDRG1 IA/ GAPDH IA. *P < 0.05.

Total RNA was extracted and the presence of NDRG1 mRNA was monitored using semiquantitative RT-PCR. Our results showed that NDRG1 mRNA expression was not amplified in 45 out of 87 breast tumors, while only 10 normal tissues lost NDRG1 expression in the corresponding normal samples. Representative data are shown in Figure 1. Thus, there was a significant difference between these two groups (*p* = 0.000).

### NDRG1 expression in breast tumor MDA-MB-231 and T47D cell lines

**Figure 2:**
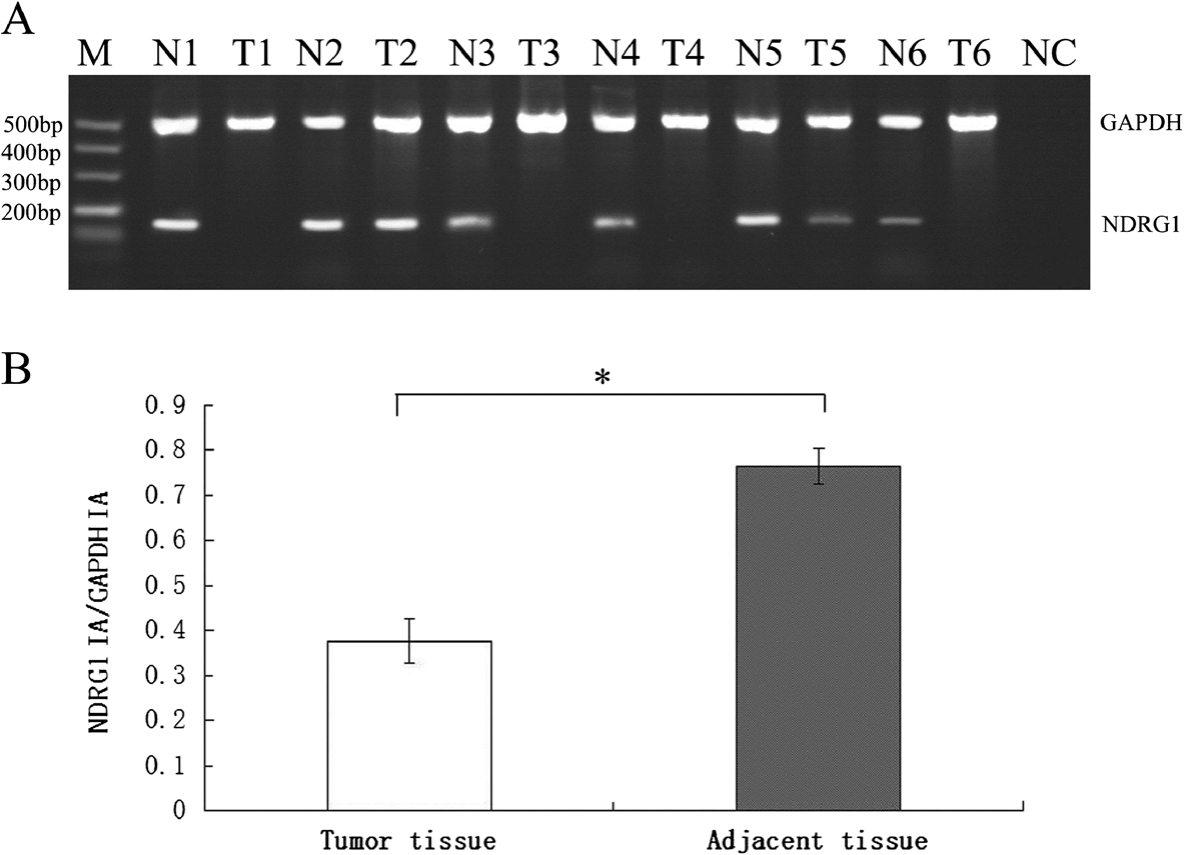
**NDRG1 expression analysis using semi-quantitative RT-PCR (A) and western blotting (B) in MDA-MB-231 (MDA) and T47D and breast cancer cell lines treated with 5-Aza-CdR for 72 h at different concentration.** The band A, B, C, D were treated with 0, 2.5, 5,10 μM of 5-Aza-CdR, respectively. NC represents the PCR reaction without DNA (negative control). The GAPDH and β-actin gene were used as a positive control for RT-PCR and western blotting respectively in this experiment. MW, Molecular Weight.

RNA and protein were extracted successfully before and after AZA treatment in MDA-MB-231 and T47D cell lines, and then was assessed with RT-PCR and western blotting, respectively. A 175 bp transcript and 43 KD protein corresponding to the NDRG1 gene were detected in the MDA-MB-231. In contrast, the T47D cell line displayed a complete absence of the NDRG1 transcript (Figure 2A).

To confirm the epigenetic transcriptional silencing of NDRG1 in breast cancer, we treated the T47D cell line with demethylating the agent AZA at 0, 2.5, 5 and 10 μM concentrations, respectively. The result showed that AZA treatment gradually restored the expression of NDRG1 in the T47D (Figure 2B).

### Silencing of NDRG1 associated with the promoter hypermethylation

**Figure 3:**
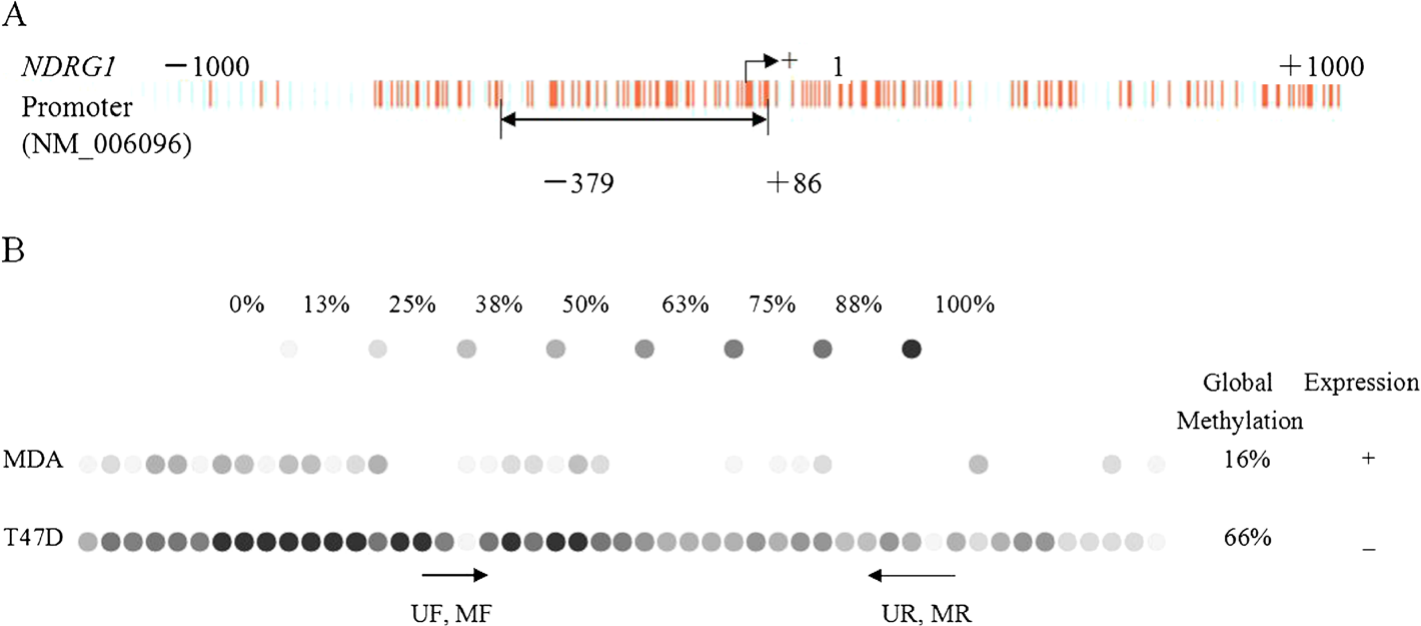
**The methylation status of NDRG1 gene in the fragment from −379 to +86. (A)** The CpG island of NDRG1 surrounding the transcription start site. An fragment of 465 bp (−379 to +86), encompassing 49 CpG dinucleotides with the CpG island was selected for methylation analysis. **(B)** The bisulfite genomic sequencing of eight individual clones was analyze in MDA-MB-231 (MDA) and T47D breast cancer cell lines. The forty-nine dinucleotides are numbered in agreement with the sequence. The open circles represent the unmethylated dinucleotides while the gray to blank portion represents the percentage of methylation. On the above side methylation pattern are represented according to data of the methylation percentage value. The arrows below the CpG dinucleotides represent the MSP primers that were used.

Sodium bisulfite sequencing was carried out on 465 bp fragment with 49 CpG dinucleotides located within the −379 to +86 island (Figure 3A). The NDRG1-negative cell line, T47D, showed a hypermethylation of 66% of the CpG dinucleotides (Figure 3B). In contrast, the cell line that expressed NDRG1, which was MDA-MB-231, had lower levels of 16% CpG dinucleotide methylation. The methylation profile comparing the NDRG1-expression cell line MDA-MB-231 to the non-expression cell line T47D demonstrated that the differentially methlylated dinucleotides were CpGs 15–18, 26–30 and 35–40. The inactivation of NDRG1 is explained through the high density of methylation in the T47D cell line (Figure 3B). This silencing due to hypermethylation was confirmed by treatment with AZA, a demethylating agent, and resulted in the subsequent expression of NDRG1 (Figure 2). As the silencing of the NDRG1 gene would be regulated by above different methylated CpGs , the regions were subsequently analyzed in primary tumors samples through nested-MSP.

### Nested-MSP analysis in breast cancer cell lines and primary breast tumors

**Figure 4:**
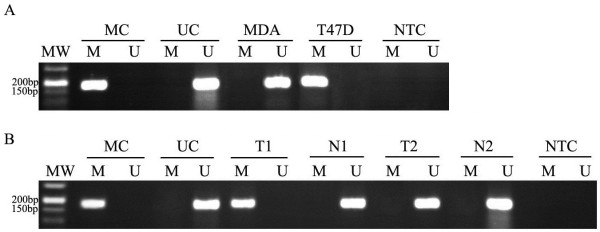
**Methylation-specific PCR analysis for NDRG1 methylation in MDA-MB-231 (MDA) and T47D breast cancer cell lines (A), breast cancer tissues (T) and corresponding normal breast tissues (N) (B).** Genomic DNA from breast cancer cell lines and breast tumors treated with sodium bisulfite were amplified using methylated (M) and unmethylated primers (U). MC was used as a positive control for methylated reaction, and UC was used as a positive control for unmethylated reaction. NTC was blank control.

To confirm our bisulfite sequencing results, we then performed nested-MSP in these two breast cancer cell lines (Figure 4A). The NDRG1 promoter of the T47D cell line consistently showed a methylated fragment. The MDA-MB-231 cell line, which express NDRG1, did not be affected after AZA treatment (Figure 1). Therefore, the MSP results of the breast tumor cell lines were proven with both the data of NDRG1 expression and sequencing data.

In this study, differentially methylated CpG island regions were subsequently used to analyze the methylation of the NDRG1 gene in primary breast cancer tumors. Of the 389 incidence breast cancer cases from a large number of populations in Qingdao, China, the methylat-ion of NDRG1 was detected in 121 cases (31.1%) showing CpG methylation in the region evaluated by nested-MSP. Two representative tumor samples are shown in Figure 4B. For 87 frozen tumor tissues, corresponding normal tissues from the same patients were obtained as well. Of 87 frozen tumors, 32 were methylated while only thirteen normal tissues appeared to be methylated. Thus, the promoter methylation of NDRG1 was observed more frequently in breast cancer tissues than the corresponding normal tissues (*P =* 0.000) (Table 1).

**Table 1:** Comparison of NDRG1 methylation between breast tumors and corresponding adjacent tissues from the same patients (n = 87)

	**NDRG1 methylation**		***P *value**
	**+**	**-**	
Tumor tissues	32	55	
Adjacent tissues	13	74	0.000

### Correlations between NDRG1 promoter methylation and its expression in breast cancer tissues

In 87 fresh breast cancer tissues, the correlation analysis revealed that NDRG1 mRNA expression loss was correlated with its methylation, and the data in Table 2 show that the difference in NDRG1 expression between methylated and unmethylated tissues was statistically significant in the samples with tumors and the corresponding normal tissues.

**Table 2:** Correlation of NDRG1 methylation and its expression

**NDRG1 methylation**	**NDRG1 expression**		**P value**
	**+**	**-**	
Tumor samples			
+	5	27	
-	37	18	0.000
Normal tissues			
+	4	9	
-	73	1	0.000

### Correlations between NDRG1 promoter methylation and the clinicopathological data

The clinical significance of the promoter methylation of the NDRG1 gene was determined by investigating the relationship between NDRG1 methylation and the clinicopathological characteristics of breast cancer patients, such as menopause, tumor grade, TNM stage, lymph node metastasis, p53 mutation, tumor size, HER2 status, and the Ki67 proliferation index. Table 3 Shows that the strongest clinicopathological characteristics are for NDRG1 methylation with histology for moderate and poor nuclear grade status [ORs:1.89 (95%CIs, 1.20-3.20) and ORs: 2.96 (95%CIs, 1.19-7.39), respectively], lymph node invasion [(ORs: 2.22 (95%CIs, 1.19-4.11)], Metastasis [(ORs: 1.94(95%CIs, 1.10-3.40)], TNM at stages III/IV [ORs: 2.82 (95%CIs, 1.41-5.62)]. These results suggested that CpG island methylation in the NDRG1 gene might be an important prognostic factor for breast cancer.

**Table 3:** Clinicopathological features of the 389 patients with primary breast carcinomas according to methylation status of NDRG1

**Clinical**	**Samples**	**NDRG1**		
**Data**	**n**	**M**	**ORs (95%CIs)**	**P value**
Menopausal status				
Pre-	162	51	1.0	
Post-	227	70	1.86(0.84-4.14)	0.126
Tumor size				
<1.0	7	3	1.0	
1.0-1.9	136	45	0.84(0.14-4.88)	0.842
2-3	200	61	0.64(0.11-3.69)	0.615
≥3.1	46	12	0.55(0.08-3.59)	0.535
Histological grade				
Well	150	35	1.0	
Moderate	207	72	**1.89(1.20-3.20)**	**0.017**
Poor	32	14	**2.96(1.19-7.39)**	**0.020**
Metastasis				
Negative	232	63	1.0	
Positive	157	58	**1.94(1.10-3.40)**	**0.021**
Lymph node invasion				
Negative	86	20	1.0	
Positive	303	101	**2.22(1.19-4.11)**	**0.011**
TNM stage				
0/I	87	20	1.0	
II	173	53	1.88(0.96-3.67)	0.064
III/IV	129	48	**2.82(1.41-5.62)**	**0.003**
p53 mutation				
Wild	160	50	1.0	
Mutant	229	71	1.04(0.64-1.70)	0.865
HER2 status				
Negative	270	86	1.0	
Positive	119	35	0.83(0.49-1.39)	0.472
Ki67 proliferation index				
<10%	93	32	1.0	
10%-32%	152	44	0.66(0.35-1.25)	0.200
≧33%	144	45	0.78(0.41-1.48)	0.441

## Discussion

In recent years, NDRG1 has been described as a potential tumor suppressor gene in various human cancers, including breast cancer [3,4,14,15]. In the present study, we first analyzed the expression of NDRG1 in 87 fresh frozen breast tumors, corresponding normal tissues and breast cancer cell lines. The results of the present study showed that in 87 breast cancer samples, NDRG1 transcripts were greatly depressed compared with corresponding normal tissues (p = 0.000). NDRG1 expression was detected in MDA-MB-231 while it was not present in the T47D cell line. It is well-known that, during carcinogenesis, a gain of methylation at the promoters of selected CpG islands and a loss of global methylation result in the silence of tumor suppressor genes [6,7,18]. Accordingly, the NDRG1 gene was revealed in the existence of CpG islands through the study of its promoter region. Therefore, treatment with AZA reversed the methylation status, which was related to the gradually increased expression of NDRG1 in T47D. These results indicate that the methylation status of the NDRG1 gene promoter is an important epigenetic mechanism regulating the level of its steady-state expression. In accordance with the NDRG1-expression of cell line, MDA-MB-231, and the non-expression of the cell line, T47D, we further investigated the NDRG1 methylation status of the region from −379 to +86 in the promoter of these two cell lines. Hypermethylation of NDRG1 was tested in T47D while the NDRG1 promoter detected the hypomethylation status in the MDA-MB-231 cell line. We presume that due to the different methylation statuses of the NDRG1 promoters in certain regions, the responses of the NDRG1 promoters in the expression/non-expression breast cancer cell lines to its expression are different. Accordingly, the variance in NDRG1 expression between the methylated and unmethylated tissues was statistically significant in the breast tumors and corresponding normal tissues (p = 0.000). Previous research has also demonstrated that NDRG1 gene reactivation in pancreatic, gastric, and colon cancer cell lines by the pharmacologic reversal of DNA methylation occurred via an indirect mechanism [11-13]. Taken together, all these results indicate that different NDRG1 promoter methylation statuses are associated with its expression.

Recent emphasis tented to identify the epigenetic change, especially DNA methylation regulate gene expression in human carcinogenesis. Kurdistani et al. reported that gene NDRG1 expression was significantly decreased in a variety of tumor cell lines and tumor tissue compared to the normal tissues [5]. The analysis on the structure of NDRG1 gene found that NDRG1 methylation status in promoter region could modulate its expression according to the CpG island in the first exon [19]. The NDRG1 gene was epigenetically regulated in pancreatic [13], colon [15]. The objective of this research was to study the potential mechanisms involved in silencing of the NDRG1 gene.

Furthermore, in order to prove that NDRG1 could be taken as a potential biomarker for breast cancer, the breast cancer tissues were analyzed further with MSP assay, and the relationship of the gene methylation status with clinicopathological data was determined. In our current study, the MSP results demonstrated that the status of methylation had a greater frequency in 87 breast tumors compared with corresponding normal tissues (*P <* 0.001), which showed that NDRG1 promoter methylation status was relative to the genetic risk variants for breast cancer.

Breast cancer is a complex and heterogeneous disease, the breast pathology includes a mount of entities for which distinctive histological patterns and different biological features exist. Patient age, tumor size, lymph invasion, histological grade, hormone receptor status and *HER2* status have been used historically to assess prognosis in women with breast cancer [20]. In our study, breast tumor samples with histology in moderate and poor grades, metastasis, TNM at stages III/IV, and lymph invasion status were found to be more frequent in methylation than with a high histological grade, no metastatic disease, TNM at stage І, and no lymph invasion, which showed that NDRG1 methylation plays an important role in the course of the histological grade, metastasis, TNM stages, and lymph invasion status for breast cancer. It is well-known that histological grade is based on cell differentiation degree and tumor cell morphology as a basis for evaluation. Meanwhile, the NDRG1 gene in various poorly differentiated cancer cells was a frequent occurrence compared to normal cells [21-23], and the differentiation stage was viewed as a crucial standard in the determination of the prognosis of breast cancer [24]. Accordingly, NDRG1 could take effect in the process of high lymph node invasion, metastasis and advanced pathological stages [25,26]. Based on the current findings about the correlation between the NDRG1 gene, differentiation stages, metastasis and lymph node invasion, we consider that NDRG1 is a newly identified differentiation-related and metastasis suppressor gene in breast cancer, and its methylation is associated with cell differentiation in the process of provoking cell tumorigenesis.

## Conclusion

In summary, this study provides new evidence that aberrant methylation and NDRG1 is involved in the tumorigenesis of breast cancer, and it could be reactivated from a silenced state by methylation. It is proposed that NDRG1 promoter demethylation and the restoration of NDRG1 expression has potential therapeutic use for breast cancer. Our findings also provide evidence that NDRG1 methylation is correlated with histological grade, metastasis, TNM stages, and lymph invasion status. However, because relatively little is known about the interrelation between these parameters, additional research is required to better understand their etiology. Nevertheless, the notion that NDRG1 could be reactivated from a silenced state by methylation might be a possible mechanism for breast cancer.

## Competing interests

The authors declare that they have no competing interests.

## Authors’ contributions

The work presented here was carried out in collaboration among all authors. LLH and LH designed the research theme and wrote the paper. LLH, MJZ, DLL, LW, YLG and DLM carried out experiments and analyzed the data. All authors read and approved the final manuscript.

## References

[B1] DeSantis C Siegel R Bandi P Jemal A Breast cancer statistics, 2011 CA Cancer J Clin 2011 61 6 409 418 21969133 10.3322/caac.20134

[B2] Van Belzen N Dinjens WN Diesveld MP Groen NA van der Made AC Nozawa Y Vlietstra R Trapman J Bosman FT A novel gene which is up-regulated during colon epithelial ell differentiation and down-regulated in colorectal neoplasms Lab Invest 1997 77 1 85 92 9251681

[B3] Bandyopadhyay S Pai SK Hirota S Hosobe S Takano Y Saito K Piquemal D Commes T Watabe M Gross SC Wang Y Ran S Watabe K Role of the putative tumor metastasis suppressor gene Drg-1 in breast cancer progression Oncogene 2004 23 33 5675 5681 10.1038/sj.onc.1207734 15184886

[B4] Bennett WP Colby TV Travis WD Borkowski A Jones RT Lane DP Metcalf RA Samet JM Takeshima Y Gu JR p53 protein accumulates frequently in early bronchial neoplasia Cancer Res 1993 53 20 4817 4822 8402667

[B5] Kurdistani SK Arizti P Reimer CL Sugrue MM Aaronson SA Lee SW Inhibition of tumor cell growth by RTP/rit42 and its responsiveness to p53 and DNA damage Cancer Res 1998 58 19 4439 4444 9766676

[B6] Clark SJ Melki J DNA methylation and gene silencing in cancer: which is the guilty party? Oncogene 2002 21 35 5380 5387 10.1038/sj.onc.1205598 12154400

[B7] Jones PA Baylin SB The epigenomics of cancer Cell 2007 128 4 683 692 10.1016/j.cell.2007.01.029 17320506 PMC3894624

[B8] Schmutte C Jones PA Involvement of DNA methylation in human carcinogenesis Biol Chem 1998 379 4–5 377 388 9628328 10.1515/bchm.1998.379.4-5.377

[B9] Leung SY Yuen ST Chung LP Chu KM Chan AS Ho JC hMLH1 promoter methylation and lack of hMLH1 expression in sporadic gastric carcinomas with high-frequency microsatellite instability Cancer Res 1999 59 1 159 164 9892201

[B10] Hu S Cheng L Wen B Large chromatin domains in pluripotent and differentiated cells Acta Biochim Biophys Sin (Shanghai) 2012 44 1 48 53 10.1093/abbs/gmr108 22194013

[B11] Fotovati A Abu-Ali S Kage M Shirouzu K Yamana H Kuwano M N-myc downstream-regulated gene 1 (Drg-1) a differentiation marker of human breast cancer Pathol Oncol Res 2011 17 3 525 533 10.1007/s12253-010-9342-y 21221878

[B12] Angst E Sibold S Tiffon C Weimann R Gloor B Candinas D Stroka D Cellular differentiation determines the expression of the hypoxia-inducible protein Drg-1 in pancreatic cancer Br J Cancer 2006 95 3 307 313 10.1038/sj.bjc.6603256 16832411 PMC2360652

[B13] Angst E Dawson DW Nguyen A Park J Go VL Reber HA Hines OJ Eibl G Epigenetic regulation affects N-myc downstream regulated gene-1 expression indirectly in pancreatic cancer cells Pancreas 2010 39 5 675 679 10.1097/MPA.0b013e3181c8b476 20173668 PMC2895953

[B14] Chang X Zhang S Ma J Li Z Zhi Y Chen J Lu Y Dai D Association of NDRG1 Gene Promoter Methylation with Reduced NDRG1 Expression in Gastric Cancer Cells and Tissue Specimens Cell Biochem Biophys 2012 66 1 93 101 23099645 10.1007/s12013-012-9457-8

[B15] Li Q Chen H Transcriptional silencing of N-Myc downstream-regulated gene 1 (Drg-1) in metastatic colon cancer cell line SW620 Clin Exp Metastasis 2011 28 2 127 135 10.1007/s10585-010-9366-4 21184144

[B16] Veronesi U Viale G Rotmensz N Goldhirsch A Rethinking TNM: breast cancer TNM classification for treatment decision-making and research Breast 2006 15 1 3 8 16473737 10.1016/j.breast.2005.11.011

[B17] Zou B Chim CS Zeng H Leung SY Yang Y Tu SP Lin MC Wang J He H Jiang SH Sun YW Yu LF Yuen ST Kung HF Wong BC Correlation between the single-site CpG methylation and expression silencing of the XAF1 gene in human gastric and colon cancers Gastroenterology 2006 131 6 1835 1843 10.1053/j.gastro.2006.09.050 17087954

[B18] Das PM Singal R DNA methylation and cancer J Clin Oncol 2004 22 22 4632 4642 10.1200/JCO.2004.07.151 15542813

[B19] Kalaydjieva L Gresham D Gooding R Heather L Baas F de Jonge R Blechschmidt K Angelicheva D Chandler D Worsley P Rosenthal A King RH Thomas PK N-myc downstream-regulated gene 1 is mutated in hereditary motor and sensory neuropathy-Lom Am J Hum Genet 2000 67 1 47 58 10.1086/302978 10831399 PMC1287101

[B20] Schnitt SJ Classification and prognosis of invasive breast cancer: from morphology to molecular taxonomy Mod Pathol 2010 23 Suppl 2 s60 s64 20436504 10.1038/modpathol.2010.33

[B21] Song Y Oda Y Hori M Kuroiwa K Ono M Hosoi F Basaki Y Tokunaga S Kuwano M Naito S Tsuneyoshi M N-myc downstream regulated gene-1/ Cap43 may play an important role in malignant progression of prostate cancer, in its close association with E-cadherin Hum Pathol 2010 41 2 214 222 10.1016/j.humpath.2009.07.011 19800102

[B22] Maruyama Y Ono M Kawahara A Yokoyama T Basaki Y Kage M Aoyagi S Kinoshita H Kuwano M Tumor growth suppression in pancreatic cancer by a putative metastasis suppressor gene Cap43/NDRG1/Drg-1 through modulation of angiogenesis Cancer Res 2006 66 12 6233 6242 10.1158/0008-5472.CAN-06-0183 16778198

[B23] Russo J Mailo D Hu YF Balogh G Sheriff F Russo IH Breast differentiation and its implication in cancer prevention Clin Cancer Res 2005 11 2 Pt 2 931s 936s 15701889

[B24] Page DL Prognosis and breast cancer. Recognition of lethal and favorable prognostic types Am J Surg Pathol 1991 15 4 334 349 10.1097/00000478-199104000-00002 2006713

[B25] Zhang SB Song SP Li B Zhou YS Zhang YD Expression of N-myc downstream-regulated gene 1 in primary gallbladder carcinoma and its MDA-MB-231with clinicopathological features and clinical outcome Med Oncol 2012 29 3 1866 1872 10.1007/s12032-011-0017-7 21735144

[B26] Jiang K Shen Z Ye Y Yang X Wang S A novel molecular marker for early detection and evaluating prognosis of gastric cancer: N-myc downstream regulated gene-1 (NDRG1) Scand J Gastroenterol 2010 45 7–8 898 908 20388062 10.3109/00365520903242580

